# The expression of apoptosis inducing factor (AIF) is associated with aging-related cell death in the cortex but not in the hippocampus in the TgCRND8 mouse model of Alzheimer’s disease

**DOI:** 10.1186/1471-2202-15-73

**Published:** 2014-06-10

**Authors:** Wenfeng Yu, Mathilde Bonnet, Mark Farso, Keran Ma, Jean-Guy Chabot, Elisabeth Martin, Alicia Torriglia, Zhizhong Guan, JoAnne McLaurin, Rémi Quirion, Slavica Krantic

**Affiliations:** 1 Key laboratory of Molecular Biology, Guiyang Medical University, Guiyang 550004, China; 2 Department of Psychiatry, Douglas Mental Health University Institute (DMHUI), McGill University, Verdun Montréal, Québec H4H 1R3, Canada; 3 Department Laboratory Medicine and Pathobiology, Faculty of Medicine, University of Toronto, Toronto, Ontario M5S 1A8, Canada; 4 Centre de Recherche des Cordeliers, UMRS872, Paris, France; 5 Department of Pathology in the Affiliated Hospital of Guiyang Medical University, Guiyang 550004, China

**Keywords:** Programmed cell death (PCD), Caspase-independent, Amyloid-beta peptide, Oxidative stress, Brain

## Abstract

**Background:**

Recent evidence has suggested that Alzheimer’s disease (AD)-associated neuronal loss may occur via the caspase-independent route of programmed cell death (PCD) in addition to caspase-dependent mechanisms. However, the brain region specificity of caspase-independent PCD in AD-associated neurodegeneration is unknown. We therefore used the transgenic CRND8 (TgCRND8) AD mouse model to explore whether the apoptosis inducing factor (AIF), a key mediator of caspase-independent PCD, contributes to cell loss in selected brain regions in the course of aging.

**Results:**

Increased expression of truncated AIF (tAIF), which is directly responsible for cell death induction, was observed at both 4- and 6-months of age in the cortex. Concomitant with the up-regulation of tAIF was an increase in the nuclear translocation of this protein. Heightened tAIF expression or translocation was not observed in the hippocampus or cerebellum, which were used as AD-vulnerable and relatively AD-spared regions, respectively. The cortical alterations in tAIF levels were accompanied by increased Bax expression and mitochondrial translocation. This effect was preceded by a significant reduction in ATP content and an increase in reactive oxygen species (ROS) production, detectable at 2 months of age despite negligible amounts of amyloid-beta peptides (Aβ).

**Conclusions:**

Taken together, these data suggest that AIF is likely to play a region-specific role in AD-related caspase-independent PCD, which is consistent with aging-associated mitochondrial impairment and oxidative stress.

## Background

Alzheimer’s disease (AD) is an age-related neurodegenerative disorder, histologically characterized by the extracellular deposition of amyloid β peptides (Aβ) and the intracellular accumulation of hyperphosphorylated tau. Numerous *in vitro* and *in vivo* studies have provided evidence for a key role of Aβ in AD [1,2]. Aβ-associated neurodegeneration involves cerebral cell death, but the underlying mechanisms remain largely unknown. Animal models of AD offer a unique opportunity to study the mechanisms involved in AD-related neurodegeneration, particularly the contribution of specific types of cell death during neuronal demise. Although neuronal death cannot be reproduced in all mouse models of AD, brain regions of some transgenic mice (e.g. PS1xAPP and TgCRND8) have been reported to demonstrate an age-dependent vulnerability, with the cortex and hippocampus being affected the earliest [3-5].

Neurodegeneration in age-associated dementia of the AD type is thought to involve neuronal cell loss by programmed cell death (PCD) [6,7]. Various caspases have been recognized as important mediators of neuronal PCD in AD [8] by classical caspase-dependent apoptosis. Several studies have shown the presence of activated caspases and the resulting caspase-cleaved substrates, including tau and amyloid precursor protein (APP) in post-mortem human AD brains and animal models [9-15]. However, accumulating evidence also points to the involvement of caspase-independent mechanisms in neuronal PCD [16-18]. In particular, apoptosis-inducing factor (AIF) is considered to play a central role among key effectors involved in caspase-independent neuronal cell death [19-21]. Consistently, we have recently reported the increased nuclear translocation of AIF in the hippocampus and cortex of post-mortem human tissues derived from AD patients [22]; this being indicative of caspase-independent PCD via AIF.

The mitochondrial flavoprotein AIF is synthesised in the cytoplasm as a ~ 67 kDa precursor. Its maturation involves a proteolytical cleavage of the precursor to a ubiquitously expressed ~ 62 kDa form [20]. Mature AIF is imbedded into the inner mitochondrial membrane where it is involved in organizing and/or maintaining the structural integrity of the respiratory chain complex-I [23,24]. Indeed, deficiency in AIF expression is associated with reduced complex-I activity and decreased ATP production [24]. Upon pathological mitochondrial permeabilization, AIF is further processed to a ~ 57 kDa truncated form (tAIF) [25,26], then released from mitochondria and translocated to the nucleus where it participates in the induction of caspase-independent PCD [27-29].

Interestingly, Harlequin (Hq) mutant mice with reduced expression of AIF display decreased oxidative phosphorylation in specific neuronal populations [24]. The reduced expression of AIF in Hq mice is correlated with a lowered expression of mitochondrial complex-I, together with signs of oxidative stress linked to increased reactive oxygen species (ROS) production in dying neurons [24,30,31]. Thus, mitochondrial dysfunction, seen in the course of rodent brain aging, is strikingly similar to that observed in AIF-deficient neurons, suggesting a possible relationship between age-related decreases in AIF expression, mitochondrial impairment and neuronal death. This hypothesis is in line with the fact that mitochondrial injury in Hq mice, due to low AIF expression, precedes the onset of neurodegeneration [32].

Moreover, although AIF is ubiquitously expressed in the rodent brain, we observed a brain region-specific gradient of distribution in the normal rat brain [33]. Such contrasting levels of AIF expression indicated that AIF-induced PCD may be region specific. To explore this possibility, we investigated AIF-related cell death in brain regions vulnerable to AD-like pathology in the transgenic mouse model, TgCRND8 (Tg), at 2, 4, 6–7 and 9 months of age. These selected ages correspond to pre-plaque, plaque burden, overt AD-like pathology and advanced AD-like pathology stage, respectively [34]. The cortex and hippocampus were compared because they are the most affected regions in Tg mice [35], whereas the cerebellum was chosen as a relatively spared control region. Overall, our data suggest that among the vulnerable brain regions, AIF is involved in AD-associated PCD in the cortex but not in the hippocampus.

## Results

### Accumulation of Aβ in the cortex and hippocampus of Tg mice is accompanied by signs of oxidative stress

We examined the Aβ load as a function of age in the cortex, hippocampus and cerebellum of Tg mice. Using brain region and age as between-subject factors, a two-way ANOVA analysis of total Aβ levels showed significant brain region and age interaction (F [6,36] = 7.604, p < 0.001). Multiple comparisons showed significant regional differences in total Aβ levels, with higher levels in the cortex compared to hippocampus (p = 0.025) and cerebellum (p = 0.001).

**Figure 1:**
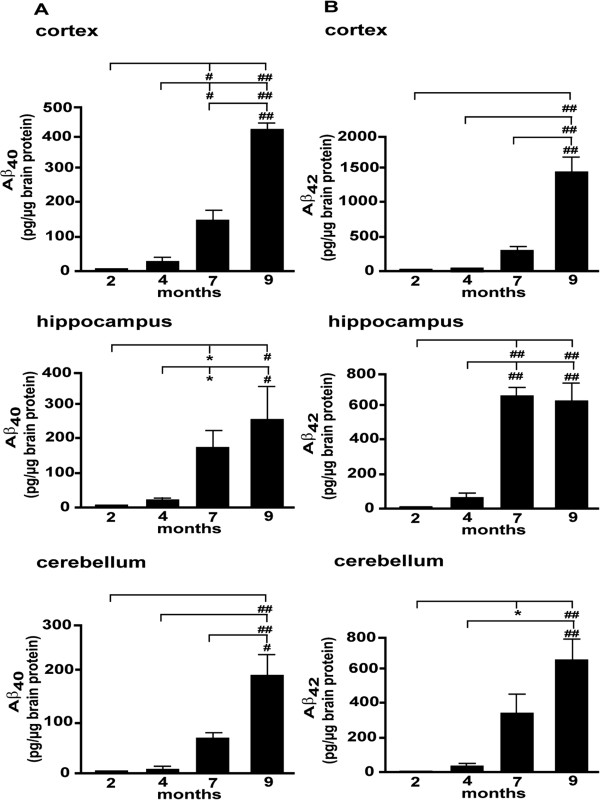
**Expression of different Aβ species in the Tg mice. (A)**: The levels of total Aβ_1–40_ were assayed by ELISA in cortical, hippocampal and cerebellar extracts of Tg mice at indicated ages. **(B)**: The levels of total Aβ_1–42_ were determined by ELISA in cortical, hippocampal and cerebellar extracts of Tg mice at indicated ages. *P < 0.05; #P < 0.01; ##P < 0.001; ###P < 0.0001.

**Figure 2:**
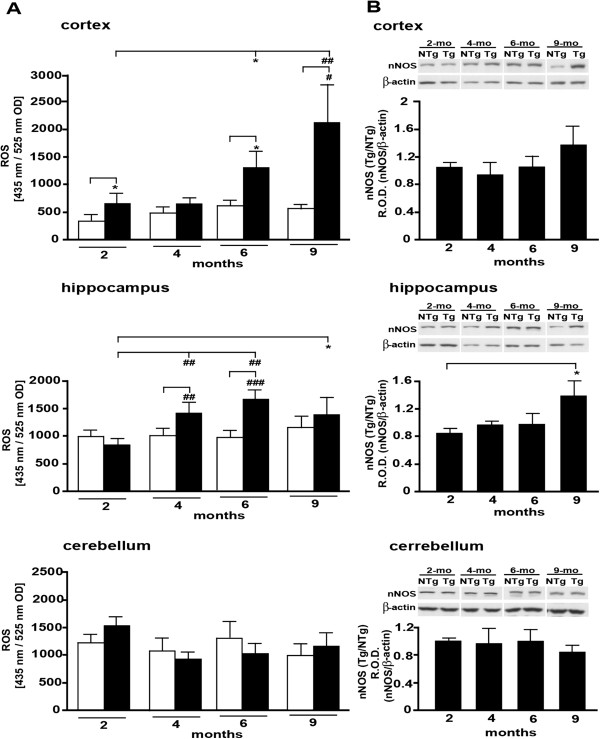
**Markers of oxidative stress in the Tg mice. (A)**: ROS expression in cortex, hippocampus and cerebellum of Tg mice as compared to their non-Tg littermates of the indicated ages. White- and black bars depict the values determined for non-Tg and Tg mice, respectively. **(B)**: nNOS quantification in cortex, hippocampus and cerebellum of Tg mice as compared to their non-Tg littermates of the indicated ages. Equal protein loading was confirmed by detection of β-actin. *P < 0.05; #P < 0.01; ##P < 0.001; ###P < 0.0001.

**Figure 3:**
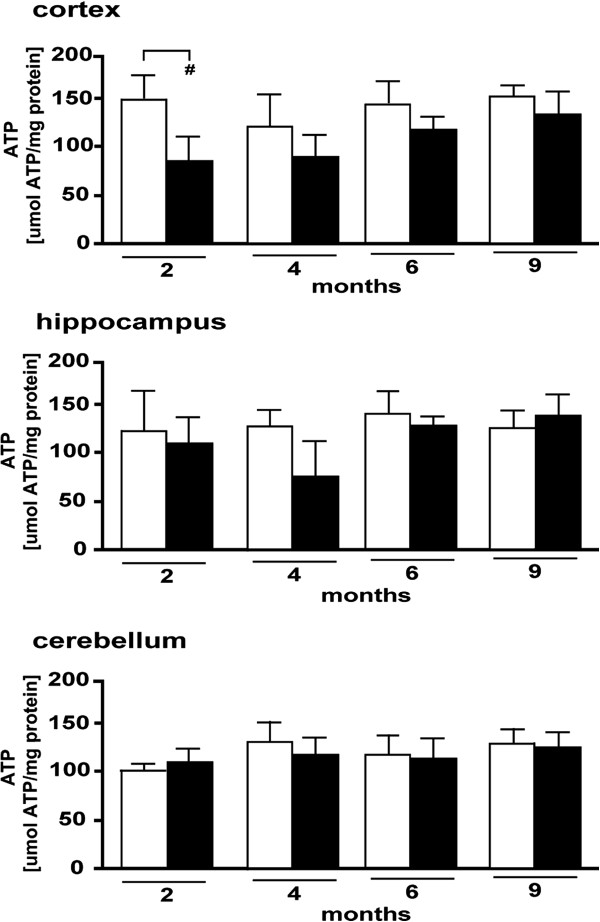
**ATP levels in the Tg mice and corresponding non**-**Tg littermates at indicated ages.** ATP is expressed in μmol ATP/mg protein. *P < 0.05; #P < 0.01; ##P < 0.001; ###P < 0.0001.

We next investigated the levels of both Aβ_1–40_ and Aβ_1–42_ as a function of age and brain region. In all three brain regions of 2- and 4-month-old mice, the level of Aβ_1–40_ (Figure 1A) and Aβ_1–42_ (Figure 1B) was low, only reaching the limit of detection at 2 months while remaining relatively low at 4 months. The difference in the level of expression of either peptide was not significant between these two ages in any of the brain regions (Figure. 1A, B). In contrast, Aβ_1–40_ (Figure 1A) and Aβ_1–42_ (Figure 1B) were significantly increased in older (7- and 9-month-old) animals. For Aβ_1–40_, the observed difference in the cortex and hippocampus was significant for 6-7-month-old animals in comparison to younger (either 2- or 4-month-old) mice. The level of Aβ_1–42_ in the hippocampus (but not in the cortex) was significantly different between 6-7-month-old mice and 2- or 4-month-old groups (Figure 1B). In all brain regions, including the cerebellum, the difference was significant for the comparison between 9-month-old animals and all other ages for both Aβ_1–40_ and Aβ_1–42_ (Figure 1A and B).Oxidative stress has been proposed to increase with Aβ peptide accumulation. We therefore undertook a parallel assessment of oxidative stress, via the quantification of ROS. The alterations seen in the cortex indicated the establishment of oxidative stress in the course of brain aging (Figure 2A). Interestingly in this region, ROS production was significantly higher in Tg than in non-Tg mice as early as 2 months of age. Moreover, the difference in ROS production between the two genotypes was significant in all age groups, except for the 4-month old mice (Figure 2A). Comparison between cortical ROS levels among transgenic animals indicated a significant increase in the course of aging (except for the 4-month-old group) when 2-month-old Tg mice were taken as a reference value (Figure 2A).In the hippocampus of Tg and non-Tg mice a significant difference in ROS levels was seen between 4- and 6-month-old groups (Figure 2A). In contrast, in 2- and 9-month-old groups, the difference between Tg and non-Tg mice was not significant (Figure 2A). However, comparing the level of ROS between the ages in transgenic animals revealed that it was significantly higher in all ages when compared to 2-month-old Tg mice (Figure 2A). No significant difference was observed between either genotype or age in the cerebellum (Figure 2A), confirming this brain region as a negative control.To further explore the establishment of oxidative stress in the course of aging in the three brain regions, we studied the level of expression of neuronal nitric oxide synthase (nNOS). A moderate increase in expression of nNOS was observed between 2- and 9-month-old mice in both cortex and hippocampus but the difference was only statistically significant in the hippocampus (Figure 2B). In contrast to the cortex and hippocampus but in agreement with ROS levels, no difference was found in the cerebellum (Figure 2B).As mitochondria are known to be causally involved in the establishment of oxidative stress and a target of Aβ toxicity, we aimed to further investigate function by comparing the level of ATP production between Tg and non-Tg mice. These experiments revealed a slightly decreased production of ATP in vulnerable areas (cortex and hippocampus) of most age groups of Tg mice versus age-matched non-Tg. However, the difference was greater at the younger stages and was only significant in the cortex of the 2-month-old group (Figure 3). All other comparisons, and specifically those for the cerebellum, indicated a similar level of ATP production between the two genotypes and age groups (Figure 3).

### Expression of cell death markers in the cortex and hippocampus of aging Tg mice

To assess neuronal death, we focused on indirect markers of cell death because it is extremely difficult to directly quantify the number of dead neurons in AD mouse models [37-39]. In agreement, our attempts to quantify neuronal death by TUNEL assay didn’t reveal significant differences between genotypes (data not shown) in spite of the fact that loss of specific sub-types of neurons has been reported by 6 months of age in TgCRND8 [3,40]. In order to assess the extent of cell death in the brain of Tg mice, we determined the levels of the universal pro-apoptotic protein, Bax, and the induction of cyclin D1, the latter is considered to be an early commitment step in neuronal cell death.

**Figure 4:**
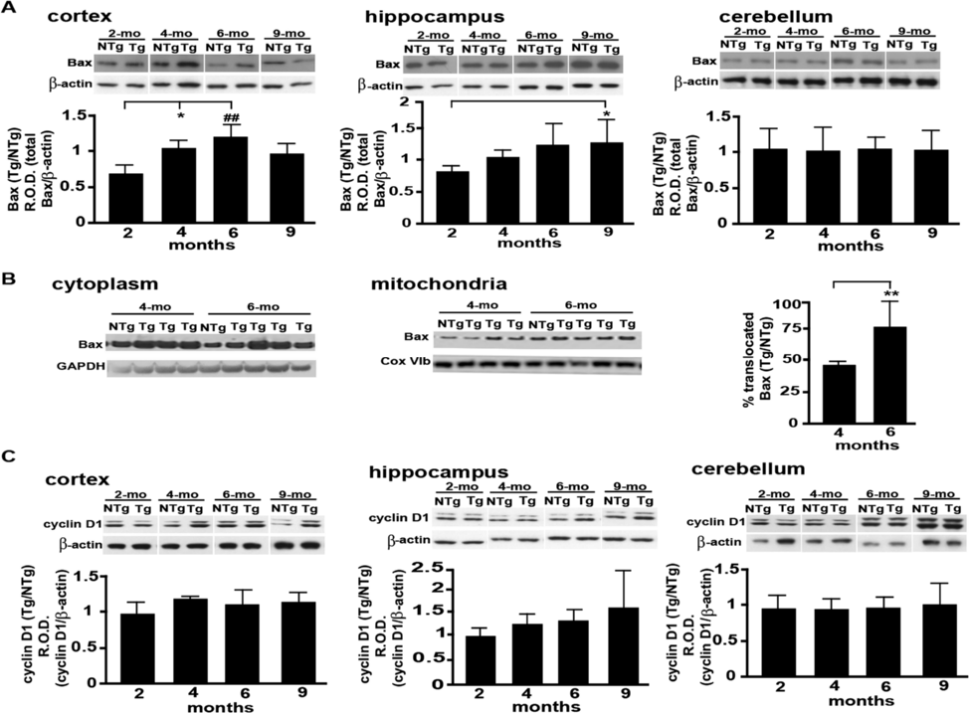
**Cell death markers expression in cortical, hippocampal and cerebellar extracts from Tg mice and age-matched non-Tg littermates. (A)**: Bax quantification in the studied brain areas as determined by Western blotting. Equal protein loading was ascertained by the parallel assessment of β-actin. **(B)**: Bax translocation as assessed by western blot probing of Bax in mitochondrial versus total Bax expression. The percentage of translocation was calculated as indicated in Materials and Methods after normalization for the equal loading over GAPDH and Cox IVb O.D. for cytoplasmic and mitochondrial fractions, respectively. **(C)**: Cyclin D1 quantification by Western blotting in the same brain regions as in **(A)**. Results are expressed as the ratio of the values calculated for the transgenic over non-Tg mice. *P < 0.05; **P < 0.01; #P < 0.01; ##P < 0.001; ###P < 0.0001.

The universal involvement of this BCl2 family member, Bax, in cell death has been largely demonstrated [41]. Moreover, Bax has recently been shown to be directly involved in Aβ toxicity to neurons [36]. In our experiments, the cortical expression of Bax is significantly increased in 4- and 6-7-month-old Tg animals when compared to the 2-month-old group (Figure 4A). Since it has been reported that gender may impact the expression of BCl2 family members and because we used sex-balanced groups of mice in our study, we investigated whether the level of Bax expression differed between males and females. We found no significant difference for either genotype (nTg p = 0.42; Tg p = 0.46). Because the death-inducing activity of Bax relies on its translocation from cytoplasm to mitochondria [41,42], we assessed the distribution of Bax between these two cellular compartments by performing the sub-cellular fractionation experiments. Our data show increased mitochondrial Bax translocation in 4- and 6 months aged TgCRND8 mice (Figure 4B), which is compatible with cell death induction. By contrast, there was no significant difference between the level of active Bax expression in cerebellum and hippocampus (Figure 4A). However, in contrast to cerebellum, hippocampus displayed an increasing, age-dependent trend of active Bax expression where only the difference between the youngest (2 months) and oldest (9 months) groups was significant (Figure 4A).

The induction of cyclin D1 is considered to precede neuronal death in some pathological paradigms. For example, cyclin D1 induction is related to excitotoxicity, which is known to be tightly associated with oxidative stress and is correlated with increasing Aβ levels [43,44]. Moreover, cyclin D1 induction has been involved in neuronal death, as shown in hippocampal [45,46] and cortical [47,48] neurons, in vitro, as well as animal and human AD post-mortem tissues [49,50]. In the current study, no difference was noted with age in any of the regions studied (Figure 4C). These results indicate that the strength of the cell death-inducing capacity of Aβ, as reflected by increased Bax levels of expression and mitochondrial translocation, which irreversibly direct cells to PCD, may be non-permissive for cyclin D1 induction. Consistently, cyclin D1 induction is generally considered as a part of the cell defence response to initially mild (reparable) DNA damage related to oxidative stress [51].

### Cell death in the cortex, but not in the hippocampus, is associated with AIF in aging Tg mice

**Figure 5:**
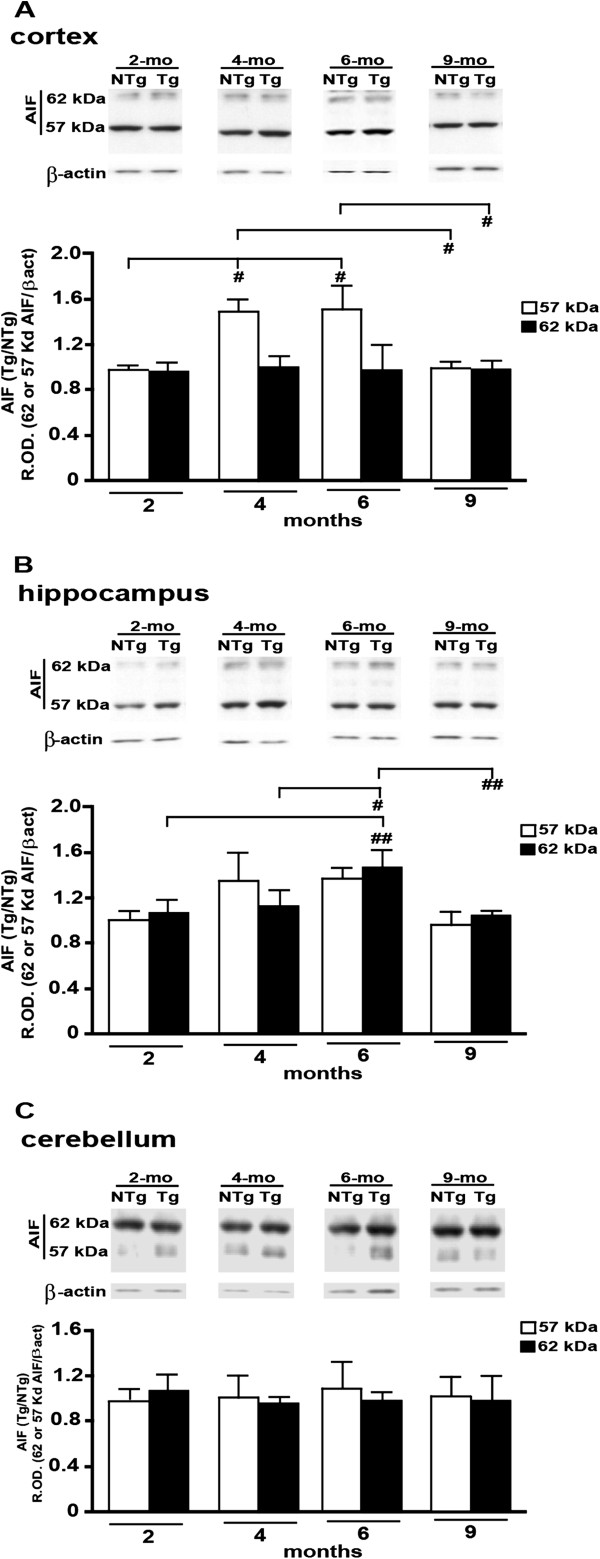
**AIF expression in cortical (A), hippocampal (B) and cerebellar (C) extracts from Tg mice and their age-matched non-Tg littermates at indicated ages.** Both mitochondrial (62 kDa, white bars) and tAIF (57 kDa, black bars) forms of AIF were studied. Equal protein loading was confirmed by detection of β-actin. Results were expressed as the ratio of the values calculated for the transgenic over non-Tg mice. *P < 0.05; #P < 0.01; ##P < 0.001; ###P < 0.0001.

The cortical expression of tAIF (57 kDa) was similar at 4- and 6-months-of-age and significantly higher than in the youngest and the oldest groups studied (Figure 5A). The expression of tAIF was not different between the age groups in either hippocampus or cerebellum (Figure 5B and 5C, respectively). Interestingly, the level of expression of the mitochondrial form (62 kDa) was significantly higher in the hippocampus of 6-month-old Tg mice compared with the other ages studied (i.e. 2, 4 and 9 months) (Figure 5B). The 62 kDa form was not altered with age in either cortex or cerebellum relative to non-Tg littermate levels (Figure 5A and 5C, respectively). However, independently of the genotype, the level of expression of the 62 kDa form appears higher in the cerebellum (Figure 5C: western blot scan) compared to the other two regions (Figure 5A and 5B: relevant western blot scans), which is in agreement with a general view that this brain region displays lower vulnerability to AD compared to the cortex and hippocampus.

**Figure 6:**
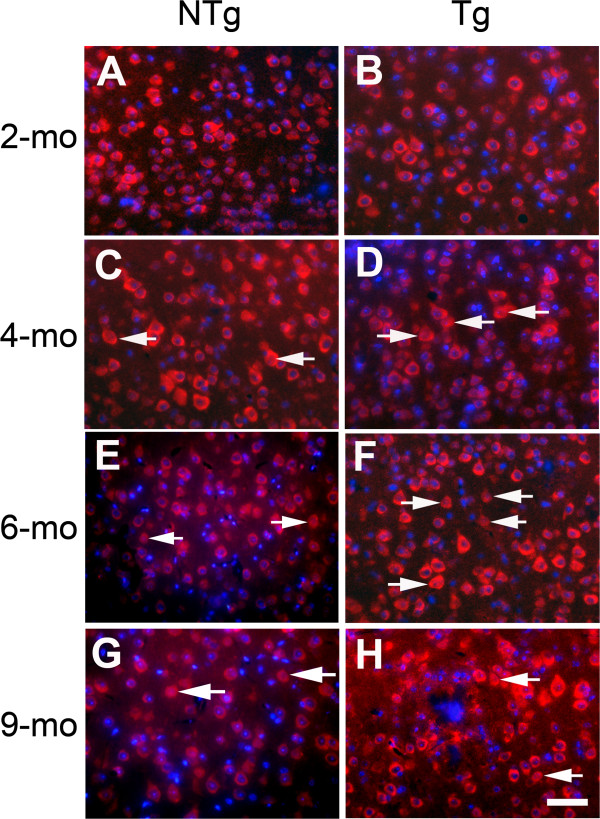
**Cellular expression of AIF in the cortex of Tg (B, D, F and H) and non-Tg littermate (A, C, E and G) mice shown at indicated ages.** Arrows point to the cells displaying the tAIF translocation to the nucleus. Bar = 30 μm.

**Figure 7:**
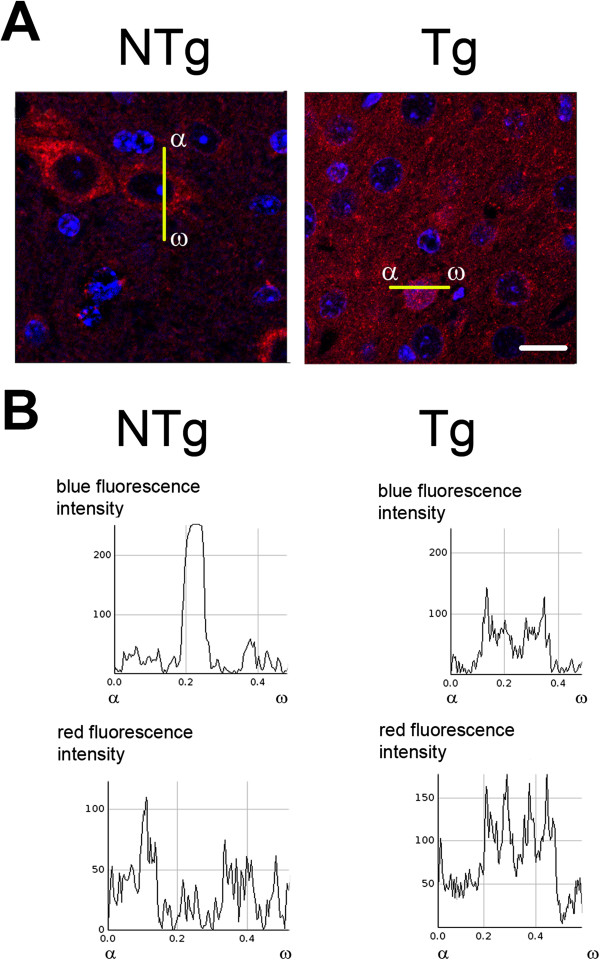
**Confocal microscopy analysis of subcellular distribution of AIF in the cortex of Tg and non-Tg littermate mice shown at indicated ages. (A)** Arrows point to the cells displaying the tAIF translocation to the nucleus which contrasts with the punctuate labelling in the cytoplasm reminiscent of mitochondrial localization. **(B)** The nuclear tranlocation of AIF was further analyzed by co-localization analysis of red and blue fluorescence corresponding to AIF and DAPI (used to stain the nuclei), respectively. Bar =10 μm.

To confirm the biochemical data pointing to the involvement of AIF in cell death in the cortex of 4- and 6-month-old Tg mice, we assessed the histological signature of AIF nuclear translocation and cell death induction [22] using immunohistochemistry. We examined the population of cells in which nuclear exclusion from AIF staining is no longer visible. In agreement with the biochemical data, a low number of cells without nuclear exclusion from AIF staining was seen in the cortex of 2-month-old mice (Figure 6A and 6B), with similar low numbers in 9-month-old mice (Figure 6G and 6H) irrespective of their genotype (non-Tg: Figure 6A and 6G and Tg: Figure 6B and 6H). In contrast, at 4 and 6–7 months of age, more cells with nuclear exclusion from AIF staining were apparent in Tg (Figure 6D and 6F) than in non-Tg (Figure 6C and 6E) mice. The occurrence of nuclear translocation of AIF has been further confirmed with higher resolution by using confocal microscopy (Figure 7). Moreover, the analysis of the co-localization profiles of AIF (red) and DAPI-stained nuclei (blue) clearly indicate the presence of AIF in the nuclear compartment of cells in which AIF has translocated to the nucleus (Figure 7B).

## Discussion

The main finding of this study showed that the cleavage of AIF into cell death-inducing tAIF increased in the course of aging and Aβ accumulation selectively in the cortex of the Tg mouse model of AD studied. Moreover, the age-dependent increase in tAIF was not found in other vulnerable (hippocampus) or relatively spared (cerebellum) brain areas further pointing to the specificity of the observed alterations in the cortex. These results are the first to indicate the contribution of the key mediator of caspase-independent programmed cell death, AIF, to the cell loss associated with age-related progression in a model of AD-like pathology.

Furthermore in our study, cortical oxidative stress in Tg mice was found to occur as early as the pre-plaque stage (i.e. 2 months), thus greatly preceding the onset of plaque burden (3–4 months) [34,40] and cell loss (6–7 months) [3,40]. These findings validate our methodological approaches, as they are in agreement with previously published data in other AD mouse models demonstrating the relationship between oxidative stress, Aβ and Aβ plaque accumulation [52-56]. Interestingly, the first signs of mitochondrial dysfunction, including signs of oxidative stress such as increased ROS production, coincide with the intracellular accumulation of Aβ and continue to accumulate with further increases in Aβ and its deposition into plaques in Thy-1 APP mice [54]. In addition, our results point to a higher Aβ load in the cortex than in the hippocampus and cerebellum of TgCRND8 mice. Moreover, despite a general trend towards the up-regulated production of ROS in both hippocampus and cortex of the youngest (2-month-old) mice, this increase was only significant in the cortex. These results are in line with the recently published data in APP/PS1 mice where oxidative stress is specifically increased in the cortex before evidence of plaque burden [53].

Besides, the increase in the level of ROS was accompanied by decreased ATP production only in the cortex of the youngest (i.e. 2 months) age group studied, further suggesting early mitochondrial dysfunction in this brain region. These results may not be surprising since creatine levels have been reported to rise with age, and this might function to buffer ATP production [57]. Remarkably, the study of the mitochondrial proteome has recently identified an early alteration in expression of more than 20 proteins, among others involved in oxidative stress and apoptosis, specifically in the cortex of another mouse model of Alzheimer’s disease [52].

The signs of mitochondrial dysfunction observed in our study preceded the increased production of tAIF and its nuclear translocation, both of which were augmented in cortices of mid-aged (4- and 6-month-old) Tg mice. Taken together, these data may suggest a causal relationship between mitochondrial impairment/oxidative stress and AIF-mediated caspase-independent PCD in this transgenic AD mouse model. In addition, these findings confirm and extend previous reports on the causal relationship between early mitochondrial dysfunction and multiple AD-associated cell death pathways including caspase-dependent apoptosis and autophagic pathways [52,56,58].

Despite the observed genotypic differences in ROS levels, the production of ATP was similar between transgenic and non-Tg mice during aging (excluding 2-month-old transgenics). Previous studies have described differences in distinct ROS scavenging capacity as well as metabolic and intracellular signalling between the cortex and hippocampus [52,55,59,60]. Therefore, the differential sensitivity of mitochondrial ATP production and increased ROS between these two regions reported herein may be associated with their respective level of expression of AIF. In agreement with this hypothesis, our previous study revealed significant differences in the level of AIF expression between brain regions of the rodent brain [33]. Furthermore, low level of AIF expression in Harlequin mice has been linked to mitochondrial respiratory chain dysfunction [24] and AIF has been implicated in ROS production [61]. Therefore, it is plausible that the tissue level of AIF may be related to the degree of Aβ-induced oxidative stress and subsequent AIF-mediated cell death.

Age-related cell death in Tg mice was demonstrated in this study by increased levels of cortical tAIF and Bax in the mid-aged groups (4- and 6-month old). However, in the oldest group (9-month-old Tg mice) AIF-mediated cell death was not evident, as indicated by the absence of increased tAIF and Bax. This apparent paradoxical observation may be related to the limited, yet still significant, neuronal loss observed in our model, starting by 6–7 months of age [3,40].

Cell death in the hippocampus has been documented at 6 months of age in TgCRND8 mice [40]. Interestingly in this study, the hippocampal level of Bax was not increased before 9 months and no significant age-dependent increase was seen in the level of tAIF, suggesting that cell death does not involve AIF and is Bax-independent, at least up to 9 months of age. In the hippocampus of the two mid-aged groups, the absence of deleterious effects (despite high levels of ROS) is suggested to be the result of compensated production of ATP, which may be related to the increased expression of the mitochondrial, 62 kDa, form of AIF. An increased expression of this form of AIF may provide more efficient respiratory chain function due to, at least in part, more efficient complex I and III function [24].

Cyclin D1 expression, used here as an early marker of neuronal cell death, was similarly expressed between different age groups in all brain regions. Although cyclin D1 induction has been associated with Aβ-induced cell death, in vitro and in human post-morten AD brain tissues [45,48-50], the data are much less conclusive regarding the relationship of cyclin D1/Aβ in AD mouse models [50,62].

## Conclusion

Overall, our data suggest that in the course of aging, AIF may play a different role in AD-related caspase-independent PCD, depending on the brain region and relative contribution of multiple PCD pathways to neurodegeneration. Since caspase-dependent apoptosis has been reported to occur both in the cortex and in the hippocampus [58], future therapeutic strategies should take into the account a multi-therapeutic approach targeting both caspase-dependent and caspase-independent PCD.

## Methods

### Reagents

Rabbit monoclonal anti-cyclin D1 was purchased from Neomarker (RM-9104-S1, Kalamazoo, MI, USA). Rabbit polyclonal anti-nNOS (sc-49055), anti-AIF (sc-9416), HRP (horseradish peroxidase)-conjugated goat-anti-rabbit IgG (sc-2054), and HRP-conjugated anti-β-actine IgG (sc-47778 HRP) were obtained from Santa Cruz (Dallas, Texas, USA). Mouse monoclonal anti-Bax (556467) was from BD Pharmingen (Qume Drive San Jose, CA, USA) whereas mouse monoclonal anti-cytochrome C (13575) and mouse monoclonal anti-COX IVb (110226) were from Abcam (Cambridge, MA, USA). Goat anti-rabbit IgGs conjugated with Alexa Fluor 568 used as a secondary antibody for immunocytochemistry was purchased from Jackson ImmunoResearch Laboratories (111-165-144, West Grove, PA, USA). Rabbit polyclonal anti-GAPDH (G9545) and all other chemicals were from Sigma-Aldrich (Oakville, Ontario, Canada).

### Animal breeding and Aβ assays in brain tissue extracts

The TgCRND8 mouse has been relatively well characterized and presents an advantage in developing AD-like pathology relatively fast. By the age of 3–4 months, these mice over-express human Aβ, encoded by a double mutated form of hAPP (695 (Swedish KM670/671NL and Indiana V717F APP mutations) transgene. Memory deficits and Aβ pathology progress in the course of aging and from 6–7 months of age, these mice have a high Aβ_42_/Aβ_40_ ratio and severe plaque load in many brain regions, including the hippocampus and cortex [34,63].

All experiments using mice followed the policies and guidelines of the Canadian Council on Animal Care, the Animal Care regulations of the University of Toronto and of McGill University on the use of laboratory animals and the U.S. National Institutes of Health Guide for the Care and Use of Laboratory Animals. Efforts were always made to minimize the numbers of animals used and their distress. Sex-balanced groups of TgCRND8 mice were maintained on an outbred C3H/C57BL6 background and kept on a 12 h light/dark cycle with food and water *ad libitum*. Four to 6 animals per experimental group were analyzed.

Brains from 2-, 4-, 6-7- and 9-month-old Tg and non-transgenic littermates (non-Tg) were dissected on ice in order to retrieve the hippocampus, cortex (to assess the vulnerable areas) and cerebellum (chosen since it is relatively spared in AD). Only Tg mice were analysed for total Aβ using a formic acid extraction procedure. Aβ_1–42_ and Aβ_1–40_ content in dissected samples were quantified with commercially available sandwich ELISA kits (Invitrogen, Burlington, ON, Canada) as described previously [63].

### ATP content

ATP content was determined in homogenates obtained from the studied brain regions by the high sensitivity ATP determination kit (Interchim, Montlucon Cedex, France) as per the manufacturer’s instructions. Briefly, brain regions were homogenized in 1 mM Tris-acetate buffer containing 2 mM EDTA and 15 μg of protein in brain homogenates was analysed. ATP disodium salt was used to prepare standard curves ranging from 10 nM to 1 mM. Luminescence was quantified by Synergy 4 (Biotek, Winooski, VT, USA) at the emission wavelength of 560 nm.

### Reactive Oxygen Species (ROS) quantification

ROS was quantified by using the conversion of non-fluorescent Dichlorodihydrofluorescein Diacetate (H_2_DCFDA) into the fluorescent 2′,7′-dichlorofluorescein (DCF) after cleavage by intracellular esterases and subsequent oxidation. In order to set up conditions for our assay, we optimized the protein concentration (50, 25 or 10 μg), incubation time (30 min, 3 h, 6 h30, 22 h and 24 h at 37°C) and presence of 50 μM H_2_0_2_. These preliminary experiments (data not shown) allowed us to determine the following experimental conditions: 20 μg of proteins per assay, 40 min incubation at 37°C and then addition of 50 μM H_2_O_2,_ followed by 5 min incubation at room temperature. The brain region homogenates used for ROS quantification were prepared in RIPA buffer as previously reported [33]. The fluorescence was quantified at the excitation/emission wavelength of 485/530 nm (Synergy 4, Biotek, Winooski, VT, USA). ROS production was expressed as a ratio of the production determined for TgCRND8 over that found for non-Tg mice within an interval of less than three days of age in order to decrease the inter-mouse variability.

### Subcellular fractionation and Bax translocation assay

Brain tissue was lysed in RIPA buffer (20 mM Tris–HCl, pH 8.0, 150 mM NaCl, 1 mM EDTA, 1% IgepalCA-630, 0.1% sodium dodecylsulfate (SDS), 50 mM NaF, 1 mM NaVO3; 2 mM phenylmethylsulfonyl fluoride, 10 μg/ml leupeptin, 50 μg/ml aprotinin) on ice for 30 min. Cell lysates were centrifuged at 15,000 × g for 15 min at 4°C twice, and the resulting supernatant, representing the cytosolic fraction, was recovered. The mitochondrial fraction was isolated with the Mitochondria Isolation Kit. Briefly, the harvested cell pellet was suspended in isolation reagent A and incubated on ice for 2 min, next the cell suspension was lysated in Dounce Tissue Grinder on ice, an equal volume of isolation reagent C was added into the cell lysate and was mixed by inverting the tube several times. After centrifugation at 700 × g for 10 min at 4°C, the supernatant was further centrifuged at 12,000 × g for 15 min at 4°C. The pellet containing the isolated mitochondria was re-suspended in RIPA buffer.

After western blot assessment (see below), the percentage of mitochondrial Bax translocation was calculated as a fraction of Bax probed in mitochondrial fraction out of total Bax (Bax probed in the mitochondrial fraction + Bax probed in the cytoplasmic fraction). The relative quantities of Bax were determined as a ratio of R.O.D measured for Bax in each cellular fraction after normalization over COX IVb and GAPDH for mitochondrial and cytoplasmic fractions, respectively. To allow the comparison between ages, the percentage of Bax translocated to mitochondria in TgCRND8 mice was normalized over the perceentage of Bax translocated to mitochondria in non-transgenic animals taken as a reference (100%).

### Western blot quantification

Protein extracts were prepared by using RIPA buffer extraction as previously reported [33]. Briefly, 40 μg of total proteins, diluted in Laemli buffer (Sigma-Aldrich, Oakville, Ontario, Canada), were boiled for 5 min and then separated by SDS-PAGE, on a Tris-Glycine 4-20% gradient gel (Invitrogen, Burlington, ON, Canada), at 130 V for 2 ½ h. Proteins were transferred to nitrocellulose membranes at 80 V for 45 min at 4°C, under agitation. Membranes were incubated with 5% skim milk diluted in Tris-buffered saline (TBS) containing 0.05% Tween (TBS-T) for 1 h at room temperature and then incubated with primary antibodies overnight at 4°C in 1% skim milk diluted in TBS-T. Three washes of 5 min in TBS-T were performed and then secondary antibodies were incubated for 1 h at room temperature in 1% milk TBS-T. After washings, the following primary antibodies were used: anti-nNOS (1:1000), anti-AIF (1: 5000), anti-cyclin D1 and anti-Bax (both in 1:500 dilution). The HRP-conjugated goat-anti-rabbit IgG was applied as a secondary antibody in parallel with the HRP-conjugated anti-β-actin IgG (both in 1:5000 dilution), used as an internal standard for equal loading. Visualization was performed using Western lightning chemiluminescence Reagent Plus kit (Perkin-Elmer, Waltham, MA, USA). Densitometric analysis of the immunoreactive bands was performed using Scion Image software (Scion Corporation, Frederick, MA, USA). The optical density (OD) measured for each immune-reactive band was normalized to β-actin. In agreement with our previous study of normal rat brain aging [33], AIF expression was relatively stable for each studied brain region in the course of non-Tg aging. Relevant AIF expression in non-Tg mice was therefore taken as a reference and results were expressed as the ratio of the values calculated for transgenic over the non-Tg mice analyzed on the same films and expressed as relative optical density (R.O.D).

### Immunohistochemistry of AIF-immunoreactive neurons in Tg mice and their non-Tg littermates

Transgenic and non-Tg littermates (age-matched at 2, 4, 6–7 and 9 months) were transcardially perfused with cold PBS-heparin and their brains isolated, post-fixed in formaldehyde and embedded in paraffin [47], and after slicing into 8 μm-thick coronal sections, immunohistochemistry proceeded. Briefly, sections were first incubated with 10% normal goat serum (NGS) in 0.05 M TBS for 1 hr at RT. For antigen retrieval, sections were incubated in 0.05 M citrate-buffered saline (pH 6.0) for 10 min at 95°C. Primary incubation followed overnight at 4°C with anti-AIF antibody in TBS (1:300 dilution). Subsequently, sections were incubated for 1 hr at RT with goat anti-rabbit IgGs conjugated with Alexa Fluor 568 (1:200) and then incubated with DAPI (2 μg/ml) for 30 min at RT. Sections were washed in TBS (3 × 5 min) between incubations. AIF immunoreactivity (AIF-ir) was labelled in red and nuclei in blue.

### Statistical analysis

GraphPad Prism 5 (GraphPad Software, San Diego, CA) or IBM SPSS Statistics 20 was used for statistical analyses. Independent t-tests were used to compare the mean group values. The mean values of three or more groups were compared with one- or two-way ANOVA and Neuman-Keuls’ post-test. In all cases, significance was noted at p < 0.05 (*), p < 0.01 (**), p < 0.001 (***). Figures were prepared with Adobe Photoshop CS4 Extended, version 11.0 (Adobe Systems Inc., San Jose, CA).

## Competing interests

The authors declare that they have no competing interests.

## Authors’ contributions

WY did the tissue microdissection, carried out western blot, immunohistochemical studies and participated in the writing of the first version of the manuscript; MB carried out ATP and ROS detection studies and analyzed the relevant data; MF did some western blot experiments, analyzed the relevant data and prepared the figures; KM performed ELISA and statistical analysis; JGC was in charge of animal breeding and genotyping; EM participated in immunohistochemical experiments; JM participated in the design and significantly contributed to the elaboration of the study; SK and RQ conceived the study and coordinated it’s realization; SK and WY wrote the first draft; AT performed confocal microscopy analysis and participated in the preparation of the figures and with ZG drafted the final version of the MS; all authors read and approved the manuscript.

## References

[B1] Hardy J Allsop D Amyloid deposition as the central event in the aetiology of Alzheimer’s disease Trends Pharmacol Sci 1991 15 383 388 1763432 10.1016/0165-6147(91)90609-v

[B2] Selkoe DJ The molecular pathology of Alzheimer’s disease Neuron 1991 15 487 498 10.1016/0896-6273(91)90052-2 1673054

[B3] Bellucci A Luccarini I Scali C Prosperi C Giovannini MG Pepeu G Casamenti F Cholinergic dysfunction, neuronal damage and axonal loss in TgCRND8 mice Neurobiol Dis 2006 15 260 272 10.1016/j.nbd.2006.03.012 16766197

[B4] Ramos B Baglietto-Vargas D del Rio JC Moreno-Gonzalez I Santa-Maria C Jimenez S Caballero C Lopez-Tellez JF Khan ZU Ruano D Gutierrez A Vitorica J Early neuropathology of somatostatin/NPY GABAergic cells in the hippocampus of a PS1xAPP transgenic model of Alzheimer’s disease Neurobiol Aging 2006 15 1658 1672 10.1016/j.neurobiolaging.2005.09.022 16271420

[B5] Schmitz C Rutten BP Pielen A Schafer S Wirths O Tremp G Czech C Blanchard V Multhaup G Rezaie P Korr H Steinbusch HW Pradier L Bayer TA Hippocampal neuron loss exceeds amyloid plaque load in a transgenic mouse model of Alzheimer’s disease Am J Pathol 2004 15 1495 1502 10.1016/S0002-9440(10)63235-X 15039236 PMC1615337

[B6] Marx J Neuroscience. New leads on the ‘how’ of Alzheimer’s Science 2001 15 2192 2194 10.1126/science.293.5538.2192 11567120

[B7] Mattson MP Apoptosis in neurodegenerative disorders Nat Rev Mol Cell Biol 2000 15 120 129 10.1038/35040009 11253364

[B8] LeBlanc AC The role of apoptotic pathways in Alzheimer’s disease neurodegeneration and cell death Curr Alzheimer Res 2005 15 389 402 10.2174/156720505774330573 16248844

[B9] Chung CW Song YH Kim IK Yoon WJ Ryu BR Jo DG Woo HN Kwon YK Kim HH Gwag BJ Mook-Jung IH Jung YK Proapoptotic effects of tau cleavage product generated by caspase-3 Neurobiol Dis 2001 15 162 172 10.1006/nbdi.2000.0335 11162250

[B10] Gamblin TC Chen F Zambrano A Abraha A Lagalwar S Guillozet AL Lu M Fu Y Garcia-Sierra F LaPointe N Miller R Berry RW Binder LI Cryns VL Caspase cleavage of tau: linking amyloid and neurofibrillary tangles in Alzheimer’s disease Proc Natl Acad Sci U S A 2003 15 10032 10037 10.1073/pnas.1630428100 12888622 PMC187753

[B11] Gervais FG Xu D Robertson GS Vaillancourt JP Zhu Y Huang J LeBlanc A Smith D Rigby M Shearman MS Clarke EE Zheng H Van Der Ploeg LH Ruffolo SC Thornberry NA Xanthoudakis S Zamboni RJ Roy S Nicholson DW Involvement of caspases in proteolytic cleavage of Alzheimer’s amyloid-beta precursor protein and amyloidogenic A beta peptide formation Cell 1999 15 395 406 10.1016/S0092-8674(00)80748-5 10319819

[B12] Guo H Albrecht S Bourdeau M Petzke T Bergeron C LeBlanc AC Active caspase-6 and caspase-6-cleaved tau in neuropil threads, neuritic plaques, and neurofibrillary tangles of Alzheimer’s disease Am J Pathol 2004 15 523 531 10.1016/S0002-9440(10)63317-2 15277226 PMC1618555

[B13] Lu DC Rabizadeh S Chandra S Shayya RF Ellerby LM Ye X Salvesen GS Koo EH Bredesen DE A second cytotoxic proteolytic peptide derived from amyloid beta-protein precursor Nat Med 2000 15 397 404 10.1038/74656 10742146

[B14] Rohn TT Head E Nesse WH Cotman CW Cribbs DH Activation of caspase-8 in the Alzheimer’s disease brain Neurobiol Dis 2001 15 1006 1016 10.1006/nbdi.2001.0449 11741396

[B15] Rohn TT Rissman RA Davis MC Kim YE Cotman CW Head E Caspase-9 activation and caspase cleavage of tau in the Alzheimer’s disease brain Neurobiol Dis 2002 15 341 354 10.1006/nbdi.2002.0549 12505426

[B16] Cregan SP Fortin A MacLaurin JG Callaghan SM Cecconi F Yu SW Dawson TM Dawson VL Park DS Kroemer G Slack RS Apoptosis-inducing factor is involved in the regulation of caspase-independent neuronal cell death J Cell Biol 2002 15 507 517 10.1083/jcb.200202130 12147675 PMC2173837

[B17] Krantic S Mechawar N Reix S Quirion R Apoptosis-inducing factor: a matter of neuron life and death Prog Neurobiol 2007 15 179 196 10.1016/j.pneurobio.2006.12.002 17267093

[B18] Lankiewicz S Marc Luetjens C Truc Bui N Krohn AJ Poppe M Cole GM Saido TC Prehn JH Activation of calpain I converts excitotoxic neuron death into a caspase-independent cell death J Biol Chem 2000 15 17064 17071 10.1074/jbc.275.22.17064 10828077

[B19] Joza N Susin SA Daugas E Stanford WL Cho SK Li CY Sasaki T Elia AJ Cheng HY Ravagnan L Ferri KF Zamzami N Wakeham A Hakem R Yoshida H Kong YY Mak TW Zúñiga-Pflücker JC Kroemer G Penninger JM Essential role of the mitochondrial apoptosis-inducing factor in programmed cell death Nature 2001 15 549 554 10.1038/35069004 11279485

[B20] Susin SA Lorenzo HK Zamzami N Marzo I Snow BE Brothers GM Mangion J Jacotot E Costantini P Loeffler M Larochette N Goodlett DR Aebersold R Siderovski DP Penninger JM Kroemer G Molecular characterization of mitochondrial apoptosis-inducing factor Nature 1999 15 441 446 10.1038/17135 9989411

[B21] Wang H Yu SW Koh DW Lew J Coombs C Bowers W Federoff HJ Poirier GG Dawson TM Dawson VL Apoptosis-inducing factor substitutes for caspase executioners in NMDA-triggered excitotoxic neuronal death J Neurosci 2004 15 10963 10973 10.1523/JNEUROSCI.3461-04.2004 15574746 PMC6730219

[B22] Yu W Mechawar N Krantic S Quirion R Evidence for the involvement of apoptosis-inducing factor-mediated caspase-independent neuronal death in Alzheimer disease Am J Pathol 2010 15 2209 2218 10.2353/ajpath.2010.090496 20228227 PMC2861086

[B23] Urbano A Lakshmanan U Choo PH Kwan JC Ng PY Guo K Dhakshinamoorthy S Porter A AIF suppresses chemical stress-induced apoptosis and maintains the transformed state of tumor cells EMBO J 2005 15 2815 2826 10.1038/sj.emboj.7600746 16001080 PMC1182241

[B24] Vahsen N Cande C Briere JJ Benit P Joza N Larochette N Mastroberardino PG Pequignot MO Casares N Lazar V Feraud O Debili N Wissing S Engelhardt S Madeo F Piacentini M Penninger JM Schägger H Rustin P Kroemer G AIF deficiency compromises oxidative phosphorylation EMBO J 2004 15 4679 4689 10.1038/sj.emboj.7600461 15526035 PMC533047

[B25] Polster BM Basanez G Etxebarria A Hardwick JM Nicholls DG Calpain I induces cleavage and release of apoptosis-inducing factor from isolated mitochondria J Biol Chem 2005 15 6447 6454 10.1074/jbc.M413269200 15590628

[B26] Yuste VJ Moubarak RS Delettre C Bras M Sancho P Robert N D’Alayer J Susin SA Cysteine protease inhibition prevents mitochondrial apoptosis-inducing factor (AIF) release Cell Death Differ 2005 15 1445 1448 10.1038/sj.cdd.4401687 15933737

[B27] Artus C Boujrad H Bouharrour A Brunelle MN Hoos S Yuste VJ Lenormand P Rousselle JC Namane A England P Lorenzo HK Susin SA AIF promotes chromatinolysis and caspase-independent programmed necrosis by interacting with histone H2AX EMBO J 2010 15 1585 1599 10.1038/emboj.2010.43 20360685 PMC2876946

[B28] Moubarak RS Yuste VJ Artus C Bouharrour A Greer PA Menissier-De Murcia J Susin SA Sequential activation of poly (ADP-ribose) polymerase 1, calpains, and Bax is essential in apoptosis-inducing factor-mediated programmed necrosis Mol Cell Biol 2007 15 4844 4862 10.1128/MCB.02141-06 17470554 PMC1951482

[B29] Yuste VJ Sanchez-Lopez I Sole C Moubarak RS Bayascas JR Dolcet X Encinas M Susin SA Comella JX The contribution of apoptosis-inducing factor, caspase-activated DNase, and inhibitor of caspase-activated DNase to the nuclear phenotype and DNA degradation during apoptosis J Biol Chem 2005 15 35670 35683 10.1074/jbc.M504015200 16049016

[B30] Klein JA Ackerman SL Oxidative stress, cell cycle, and neurodegeneration J Clin Invest 2003 15 785 793 10.1172/JCI200318182 12639981 PMC153779

[B31] Klein JA Longo-Guess CM Rossmann MP Seburn KL Hurd RE Frankel WN Bronson RT Ackerman SL The harlequin mouse mutation downregulates apoptosis-inducing factor Nature 2002 15 367 374 10.1038/nature01034 12353028

[B32] El Ghouzzi V Csaba Z Olivier P Lelouvier B Schwendimann L Dournaud P Verney C Rustin P Gressens P Apoptosis-inducing factor deficiency induces early mitochondrial degeneration in brain followed by progressive multifocal neuropathology J Neuropathol Exp Neurol 2007 15 838 847 10.1097/NEN.0b013e318148b822 17805014

[B33] Yu W Gubkina O Mechawar N Elwell D Quirion R Krantic S Expression of apoptosis-inducing factor (AIF) in the aged rat brain Neurobiol Aging 2011 15 179 180 10.1016/j.neurobiolaging.2009.01.010 19251341

[B34] Chishti MA Yang DS Janus C Phinney AL Horne P Pearson J Strome R Zuker N Loukides J French J Turner S Lozza G Grilli M Kunicki S Morissette C Paquette J Gervais F Bergeron C Fraser PE Carlson GA George-Hyslop PS Westaway D Early-onset amyloid deposition and cognitive deficits in transgenic mice expressing a double mutant form of amyloid precursor protein 695 J Biol Chem 2001 15 21562 21570 10.1074/jbc.M100710200 11279122

[B35] Salek RM Xia J Innes A Sweatman BC Adalbert R Randle S McGowan E Emson PC Griffin JL A metabolomic study of the CRND8 transgenic mouse model of Alzheimer’s disease Neurochem Int 2010 15 937 947 10.1016/j.neuint.2010.04.001 20398713

[B36] Kudo W Lee HP Smith MA Zhu X Matsuyama S Lee HG Inhibition of Bax protects neuronal cells from oligomeric Abeta neurotoxicity Cell Death Dis 2012 15 e309 10.1038/cddis.2012.43 22592316 PMC3366077

[B37] Dodart JC Mathis C Bales KR Paul SM Does my mouse have Alzheimer’s disease? Genes Brain Behav 2002 15 142 155 10.1034/j.1601-183X.2002.10302.x 12884970

[B38] Duyckaerts C Potier MC Delatour B Alzheimer disease models and human neuropathology: similarities and differences Acta Neuropathol 2008 15 5 38 18038275 10.1007/s00401-007-0312-8 PMC2100431

[B39] Janus C Westaway D Transgenic mouse models of Alzheimer’s disease Physiol Behav 2001 15 873 886 10.1016/S0031-9384(01)00524-8 11566220

[B40] Krantic S Isorce N Mechawar N Davoli MA Vignault E Albuquerque M Chabot JG Moyse E Chauvin JP Aubert I McLaurin J Quirion R Hippocampal GABAergic neurons are susceptible to amyloid-beta toxicity in vitro and are decreased in number in the Alzheimer’s disease TgCRND8 mouse model J Alzheimers Dis 2012 15 293 308 22232004 10.3233/JAD-2011-110830

[B41] Renault TT Manon S Bax: Addressed to kill Biochimie 2011 15 1379 1391 10.1016/j.biochi.2011.05.013 21641962

[B42] Renault TT Chipuk JE Death upon a kiss: mitochondrial outer membrane composition and organelle communication govern sensitivity to BAK/BAX-dependent apoptosis Chem Biol 2014 15 114 123 10.1016/j.chembiol.2013.10.009 24269152 PMC3947007

[B43] Butterfield DA Amyloid beta-peptide (1–42)-induced oxidative stress and neurotoxicity: implications for neurodegeneration in Alzheimer’s disease brain. A review Free Radic Res 2002 15 1307 1313 10.1080/1071576021000049890 12607822

[B44] Nguyen D Alavi MV Kim KY Kang T Scott RT Noh YH Lindsey JD Wissinger B Ellisman MH Weinreb RN Perkins GA Ju WK A new vicious cycle involving glutamate excitotoxicity, oxidative stress and mitochondrial dynamics Cell Death Dis 2011 15 e240 10.1038/cddis.2011.117 22158479 PMC3252734

[B45] Efthimiadi L Farso M Quirion R Krantic S Cyclin D1 induction preceding neuronal death via the excitotoxic NMDA pathway involves selective stimulation of extrasynaptic NMDA receptors and JNK pathway Neurodegener Dis 2012 15 80 91 10.1159/000335911 22354185

[B46] Hardingham GE Bading H Synaptic versus extrasynaptic NMDA receptor signalling: implications for neurodegenerative disorders Nat Rev Neurosci 2010 15 682 696 10.1038/nrn2911 20842175 PMC2948541

[B47] Malik B Currais A Soriano S Cell cycle-driven neuronal apoptosis specifically linked to amyloid peptide Abeta1-42 exposure is not exacerbated in a mouse model of presenilin-1 familial Alzheimer’s disease J Neurochem 2008 15 912 916 10.1111/j.1471-4159.2008.05446.x 18466334

[B48] Wu Q Combs C Cannady SB Geldmacher DS Herrup K Beta-amyloid activated microglia induce cell cycling and cell death in cultured cortical neurons Neurobiol Aging 2000 15 797 806 10.1016/S0197-4580(00)00219-0 11124423

[B49] Hoozemans JJ Bruckner MK Rozemuller AJ Veerhuis R Eikelenboom P Arendt T Cyclin D1 and cyclin E are co-localized with cyclo-oxygenase 2 (COX-2) in pyramidal neurons in Alzheimer disease temporal cortex J Neuropathol Exp Neurol 2002 15 678 688 12152783 10.1093/jnen/61.8.678

[B50] Malik B Currais A Andres A Towlson C Pitsi D Nunes A Niblock M Cooper J Hortobagyi T Soriano S Loss of neuronal cell cycle control as a mechanism of neurodegeneration in the presenilin-1 Alzheimer’s disease brain Cell Cycle 2008 15 637 646 10.4161/cc.7.5.5427 18239458

[B51] Yang Y Herrup K Cell division in the CNS: protective response or lethal event in post-mitotic neurons? Biochim Biophys Acta 2007 15 457 466 10.1016/j.bbadis.2006.10.002 17158035 PMC2785903

[B52] Chou JL Shenoy DV Thomas N Choudhary PK Laferla FM Goodman SR Breen GA Early dysregulation of the mitochondrial proteome in a mouse model of Alzheimer’s disease J Proteomics 2011 15 466 479 10.1016/j.jprot.2010.12.012 21237293

[B53] Hamilton A Holscher C The effect of ageing on neurogenesis and oxidative stress in the APP (swe)/PS1 (deltaE9) mouse model of Alzheimer’s disease Brain Res 2012 15 83 93 22418058 10.1016/j.brainres.2012.02.015

[B54] Hauptmann S Scherping I Drose S Brandt U Schulz KL Jendrach M Leuner K Eckert A Muller WE Mitochondrial dysfunction: an early event in Alzheimer pathology accumulates with age in AD transgenic mice Neurobiol Aging 2009 15 1574 1586 10.1016/j.neurobiolaging.2007.12.005 18295378

[B55] Lee SH Kim KR Ryu SY Son S Hong HS Mook-Jung I Ho WK Impaired short-term plasticity in mossy fiber synapses caused by mitochondrial dysfunction of dentate granule cells is the earliest synaptic deficit in a mouse model of Alzheimer’s disease J Neurosci 2012 15 5953 5963 10.1523/JNEUROSCI.0465-12.2012 22539855 PMC6703608

[B56] Reddy PH McWeeney S Park BS Manczak M Gutala RV Partovi D Jung Y Yau V Searles R Mori M Quinn J Gene expression profiles of transcripts in amyloid precursor protein transgenic mice: up-regulation of mitochondrial metabolism and apoptotic genes is an early cellular change in Alzheimer’s disease Hum Mol Genet 2004 15 1225 1240 10.1093/hmg/ddh140 15115763

[B57] Kuzyk A Kastyak M Agrawal V Gallant M Sivakumar G Rak M Del Bigio MR Westaway D Julian R Gough KM Association among amyloid plaque, lipid, and creatine in hippocampus of TgCRND8 mouse model for Alzheimer disease J Biol Chem 2010 15 31202 31207 10.1074/jbc.M110.142174 20682779 PMC2951194

[B58] Yang DS Kumar A Stavrides P Peterson J Peterhoff CM Pawlik M Levy E Cataldo AM Nixon RA Neuronal apoptosis and autophagy cross talk in aging PS/APP mice, a model of Alzheimer’s disease Am J Pathol 2008 15 665 681 10.2353/ajpath.2008.071176 18688038 PMC2527090

[B59] Cimini A Moreno S D’Amelio M Cristiano L D’Angelo B Falone S Benedetti E Carrara P Fanelli F Cecconi F Amicarelli F Cerù MP Early biochemical and morphological modifications in the brain of a transgenic mouse model of Alzheimer’s disease: a role for peroxisomes J Alzheimers Dis 2009 15 935 952 19749434 10.3233/JAD-2009-1199

[B60] Fenili D Weng YQ Aubert I Nitz M McLaurin J Sodium/myo-Inositol transporters: substrate transport requirements and regional brain expression in the TgCRND8 mouse model of amyloid pathology PLoS One 2011 15 e24032 10.1371/journal.pone.0024032 21887366 PMC3162603

[B61] Chinta SJ Rane A Yadava N Andersen JK Nicholls DG Polster BM Reactive oxygen species regulation by AIF- and complex I-depleted brain mitochondria Free Radic Biol Med 2009 15 939 947 10.1016/j.freeradbiomed.2009.01.010 19280713 PMC2775507

[B62] Gartner U Bruckner MK Krug S Schmetsdorf S Staufenbiel M Arendt T Amyloid deposition in APP23 mice is associated with the expression of cyclins in astrocytes but not in neurons Acta Neuropathol 2003 15 535 544 10.1007/s00401-003-0760-8 12923647

[B63] Ma K Mount HT McLaurin J Region-specific distribution of beta-amyloid peptide and cytokine expression in TgCRND8 mice Neurosci Lett 2011 15 5 10 10.1016/j.neulet.2011.01.035 21295112

